# Supplementary material for: Ultrathin 2 nm gold as impedance-matched absorber for infrared light
Source: Nat Commun. 2020 May 1;11:2161. doi: 10.1038/s41467-020-15762-3 (PMC7195431; doi:10.1038/s41467-020-15762-3)
Supplement: Supplementary file 1 — Supplementary Information [file 41467_2020_15762_MOESM1_ESM.pdf]

Supplementary Information:
Ultrathin 2 nm gold as impedance-matched absorber for infrared light

Niklas Luhmann,[1] Dennis Høj,[2] Markus Piller,[1] Hendrik Kähler,[1] Miao-Hsuan Chien,[1] Robert G. West,[1] Ulrik Lund Andersen,[2] and Silvan Schmid[1]

[1]*Institute of Sensor and Actuator Systems, TU Wien, 1040 Vienna, Austria.*

[2]*Department of Physics, Technical University of Denmark, 2800 Kongens Lyngby, Denmark*

SUPPLEMENTARY METHODS: OPTICAL MODEL

In order to predict the optical properties as transmittance and reflectivity, a general matrix method based on [1] has been applied. The incident light is assumed to be non-polarized and normal to the metal surface. The material layers are assumed to be coherent, e.g. isotropic and homogeneous. Assuming all copper has been oxidized, hence not conductive, it will have negligible contribution and is, therefore, excluded from the model. Optical data for Si_xN_y and Au are extracted experimentally which will be described in the following section.

With the assumptions mentioned in the text the multi-layer membrane can optically be described as

$$\begin{bmatrix} E_{0\mathrm{R}}^{+} \\ E_{0\mathrm{R}}^{-} \end{bmatrix} = \mathbf{S} \begin{bmatrix} E_{(n+1)\mathrm{L}}^{+} \\ E_{(n+1)\mathrm{L}}^{-} \end{bmatrix} , \tag{1}$$

where E_i^+ and E_i^- is the electric field associated with a positive and negative going direction, respectively. The subscript i indicates the E-field's position in the layered system with 0R and $(k+1)$L being just before the first layer and just after the last layer, respectively. $\mathbf{S}$ is the system matrix. For a vacuum-Au-Si_xN_y-vacuum system using the subscripts v, m, d, v, respectively, this is defined as

$$\mathbf{S} = \mathbf{I}_{\mathrm{vm}}\mathbf{L}_{\mathrm{m}}\mathbf{I}_{\mathrm{md}}\mathbf{L}_{\mathrm{d}}\mathbf{I}_{\mathrm{dv}} , \tag{2}$$

where $\mathbf{I}_{ij}$ and $\mathbf{L}_j$ describes the wave propagation through the interface and film, respectively, and defined as

$$\mathbf{I}_{ij} = \frac{1}{t_{ij}} \begin{bmatrix} 1 & r_{ij} \\ r_{ij} & 1 \end{bmatrix} \tag{3}$$

$$\mathbf{L}_j = \begin{bmatrix} \exp(-\mathrm{i}\beta_j) & 0 \\ 0 & \exp(\mathrm{i}\beta_j) \end{bmatrix} , \tag{4}$$

and

$$r_{ij} = \frac{N_j - N_i}{N_j + N_i} \quad t_{ij} = \frac{2N_i}{N_j + N_i} \quad \beta_j = \frac{2\pi d_j N_j}{\lambda} , \tag{5}$$

where N_i is the complex refractive index of a material layer, d_j its thickness, and λ is the optical wavelength in free space. From $\mathbf{S}$ it is possible to estimate the overall reflection and

transmission coefficients $r = S_{21}/S_{11}$ and $t = 1/S_{11}$, respectively, from which the system's reflectivity R and transmittance T are defined as

$$R = \left|r^2\right| \quad , \quad T = \left|t^2\right| \ . \tag{6}$$

Note that these equations are only valid with the assumptions given in the text and using vacuum as the first and final material layer. For a system only containing Si_xN_y, Eq. (2) reduces to

$$\mathbf{S} = \mathbf{I}_{\mathrm{vd}}\mathbf{L}_{\mathrm{d}}\mathbf{I}_{\mathrm{dv}} \ . \tag{7}$$

SUPPLEMENTARY NOTE 1: COMPARISON WITH IMPEDANCE-MATCH THEORY

For a direct comparison of the deposited gold films to the impedance-match theory introduced by W. Woltersdorff [2], the average measured transmittance and reflectivity of each film is plotted as function of the sheet resistance. In order to avoid the additional absorption dip of the supporting Si_xN_y, the spectral average was taken from 15 µm to 20 µm. The theoretic reflectivity R, transmittance T and absorptivity A are then given by Eq. (15), (17) and (18) in [3]:

$$T = 4/D \tag{8}$$

$$A = 4f/D \tag{9}$$

$$R = 1 - T - A \tag{10}$$

$$D = n^2 \left[\frac{f+1}{n^2} + 1\right] \sin^2 kd + (f+2)^2 \cos^2 kd \tag{11}$$

where $f = Z_0/R_\mathrm{S}$ is a ratio of the free space impedance and sheet resistance of the UTMF, n, k the refractive index and wavenumber of the dielectric support (Si_xN_y), with a total thickness of $d = 50\,\mathrm{nm}$. The optical constants n, k of the used Si_xN_y are taken from the fit in figure 3 of the manuscript. The results are presented in figure 1. While the percolated,

conducting films can be well described by the model, thinner films in the insulating region (blue) strongly deviate. The transition limit at approximately $3.2 \times 10^3\,\Omega$ was estimated by the fitted percolation threshold of 1.84 nm and the limiting resistivity of $6\,\mu\Omega \cdot \mathrm{m}$, defined by Ioffe-Regel [4]. In comparison, this deviation of the resistivity and sheet resistance below percolation can be also observed in fig. 2a,b. The origin of this offset can be mainly related to a systematic error since the used four-point-probe setup could not provide more precise values below the insulator-to-conductor transition.

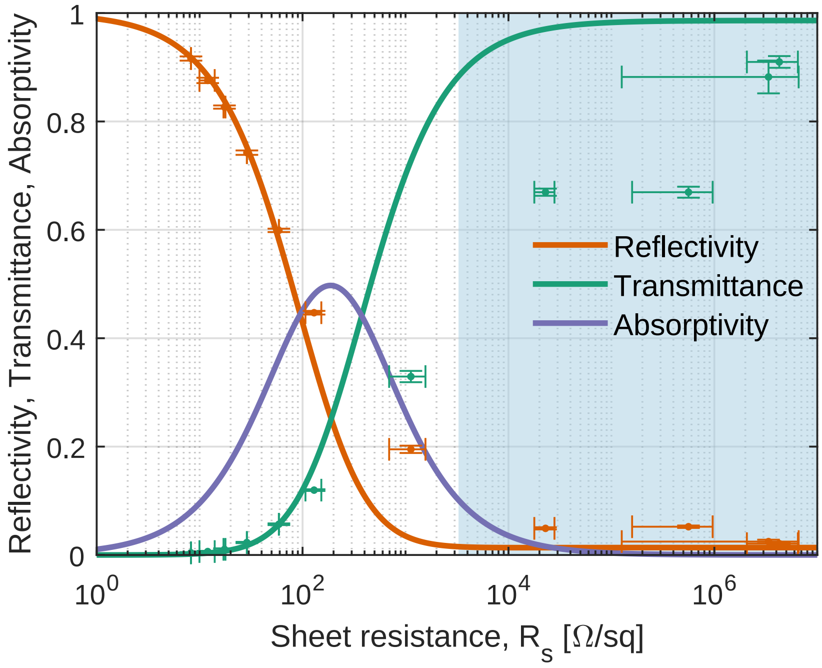

FIG. 1. Comparison to impedance-match theory. Average transmittance and reflectivity from 15 µm to 20 µm of all fabricated Au films as function of the sheet resistance: Dots represent the measured data, solid lines are calculated by theory Eqs. (8)–(11), using the extracted optical properties of the supporting dielectric (Si_xN_y). For (percolated) conducting films the data can be well described by the model, below percolation (blue zone), measured values strongly vary due to the transition to an insulating surface. Error bars resemble the standard deviation from average based on multiple individual measurements for samples between 0.35 nm to 3.0 nm and else single measurements. Source data are provided as a Source Data file.

SUPPLEMENTARY NOTE 2: UTMF MORPHOLOGY

Figure 2 shows exemplary surface scans of a bare Si_xN_y membrane (a) and the 2 nm UTMF absorber (b), recorded with an atomic force microscope (AFM) Bruker Dimension

Edge. As can be observed, the UTMF surface appears in general smooth and without confined grains, which supports the conclusion of an uniform metal film. In comparison, the bare supporting Si_xN_y showed a rougher surface. A typical way to access the uniformity is the evaluation of the root mean square roughness R_q. For the demonstrated scans we found a slight reduction from the bare Si_xN_y with $R_q = 23.47$ pm down to 19.18 pm for the UTMF, which is far below the total deposited gold thickness of 2 nm. In addition, modeling of the optical constants of the UTMF using an effective medium approximation resulted in a worse match of the measured properties when compared to treating the layer as an uniform Drude-like metal. Thus, the fabricated absorber can be seen as an uniform and continuous metal film.

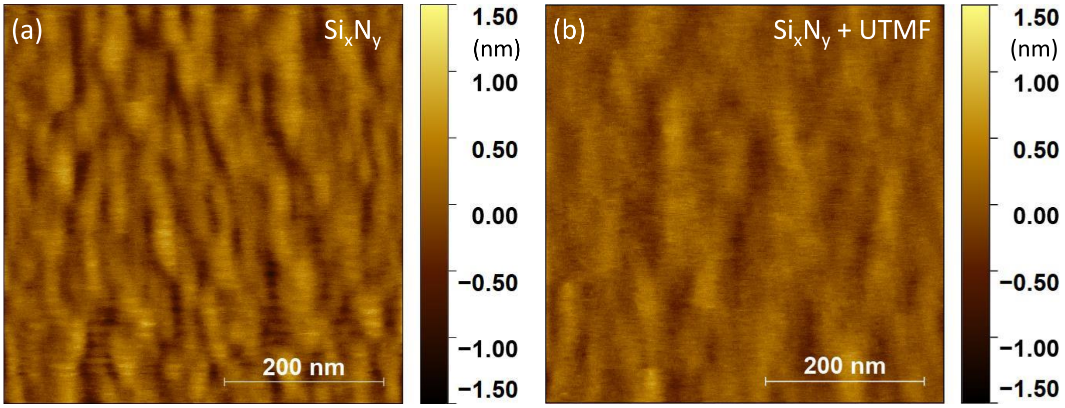

FIG. 2. AFM analysis of the morphology. (a) Exemplary bare Si_xN_y membrane (b) UTMF absorber made of 2 nm Au on top of oxidized copper. The UTMF in general shows a smooth and uniform surface supporting the conclusion of a continuous metal film. The resulting root mean square roughness in addition shows a reduction from $R_q = 23.47$ pm of the bare Si_xN_y to 19.18 pm for the UTMF. Source data are provided as a Source Data file.

SUPPLEMENTARY NOTE 3: RESISTIVITY BELOW PERCOLATION

As can be seen in Fig. 2 of the manuscript, the resistivity of seeded gold layers below percolation show an unexpected reverse trend for the thinnest films. Previous studies confirm that this feature can be related to the growth of Au islands, which, due to the increased surface area, act as additional scattering centres, causing an increase in resistivity [5, 6]. For a more detailed investigation, additional 4-point-probe (4PP) measurements were made on all samples below percolation (0.35 nm to 1.5 nm). Figure 3 (a) shows the obtained resistivities as a function of probing current. One feature that can be seen is that these

unpercolated layers show a strong drop in resistivity for higher probing currents. This effect can be attributed to thermal heating caused by the probing current. Therefore, only the lower probe current range (marked in orange) was taken for subsequent statistical data analysis. Figure 3 (b) shows the resulting average resistivties in comparison to measured data extracted from Grunznev et al. [5]. As can be seen in both data sets, the resistivity follows the same reverse trend for Au films below approximately 1.0 nm.

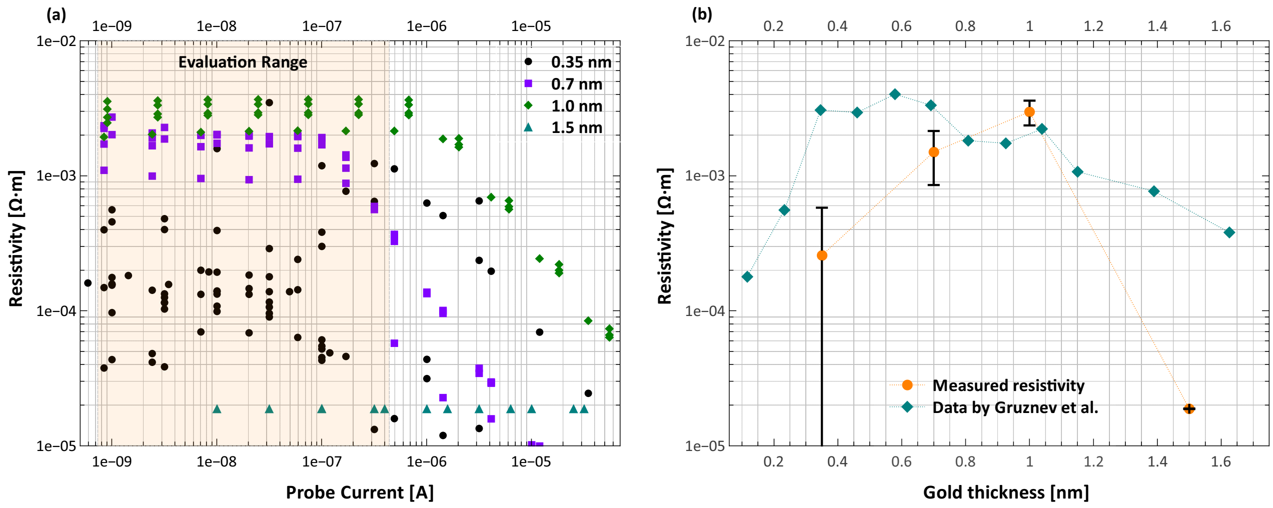

FIG. 3. UTMF resistivity below percolation. (a) Additional 4PP measurements of UTMF samples. For the statistical analysis following independent measurements were taken on each single sample: 0.35 nm – 11x ; 0.7 nm – 6x ; 1.0 nm – 5x ; 1.5 nm – 2x. (b) Comparison of extracted average resistivities to a previous study of UTMF on seeded surfaces by Grunznev et al. [5]. The error bars represent the 95 % confidence interval. Source data are provided as a Source Data file.

---

[1] E. Centurioni, Generalized matrix method for calculation of internal light energy flux in mixed coherent and incoherent multilayers, Applied Optics 44, 7532–7539, doi:10.1364/AO.44.007532 (2005).

[2] W. Woltersdorff, Über die optischen Konstanten dünner Metallschichten im langwelligen Ultrarot, Zeitschrift für Physik **91**, 230–252 (1934).

[3] C. Hilsum, Infrared Absorption of Thin Metal Films at Non-Normal Incidence, Journal of the Optical Society of America **45**, 135–136 (1955).

[4] A.F. Ioffe and A.R. Regel, Non-crystalline, amorphous and liquid electronic semiconductors, Prog. Semicond **4**, 237f. (1960).

[5] D. V. Gruznev, D. A. Olyanich, D. N. Chubenko, D. A. Tsukanov, E. A. Borisenko, L. V. Bondarenko, M. V. Ivanchenko, A. V. Zotov, and A. A. Saranin, Growth of Au thin film on Cu-modified Si(1 1 1) surface, Surface Science **603**, 24, 3400–3403 (2009).

[6] Z. Korczak and T. Kwapiński, Electrical conductance at initial stage in epitaxial growth of Pb, Ag, Au and In on modified Si (1-1-1) surface, Surface Science **601**, 16, 3324–3334 (2007).
