# Supplementary figures and images for: Ultrathin 2 nm gold as impedance-matched absorber for infrared light
Source: Nat Commun. 2020 May 1;11:2161. doi: 10.1038/s41467-020-15762-3 (PMC7195431; doi:10.1038/s41467-020-15762-3)

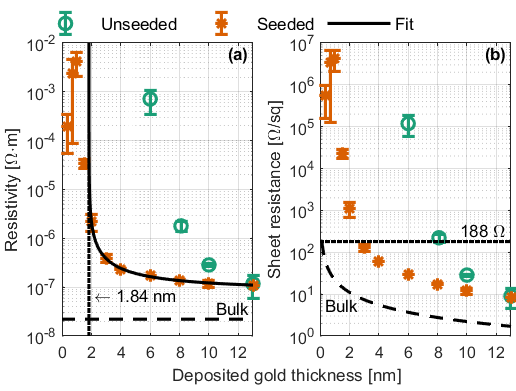

Supplement: Supplementary file 3 — Source Data [file 41467_2020_15762_MOESM3_ESM.zip › SOURCE DATA/Fig.2 - Resistivity data and dataprocessing/R_vs_h_1.png]

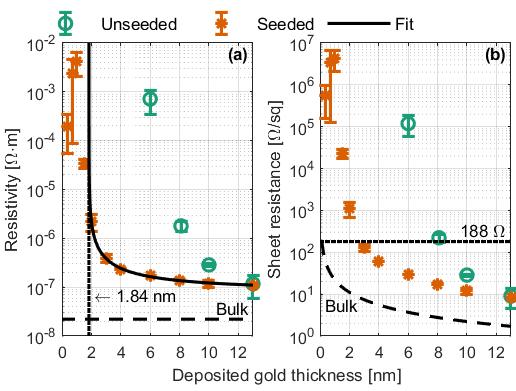

Supplement: Supplementary file 3 — Source Data [file 41467_2020_15762_MOESM3_ESM.zip › SOURCE DATA/Fig.2 - Resistivity data and dataprocessing/R_vs_h_2.png]

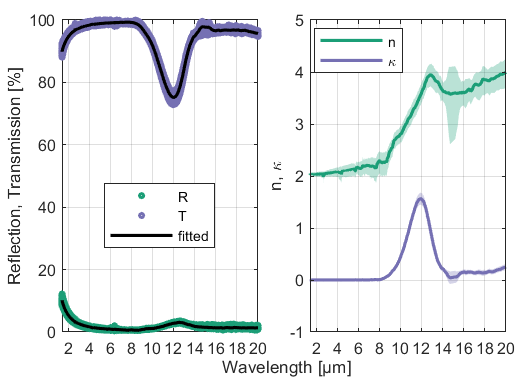

Supplement: Supplementary file 3 — Source Data [file 41467_2020_15762_MOESM3_ESM.zip › SOURCE DATA/Fig.3 - SiN optical measurements and dataprocessing/SiN_fit.png]

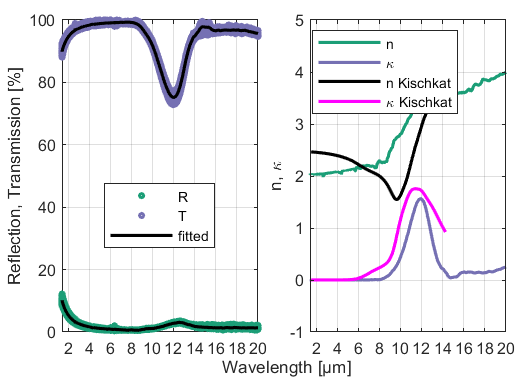

Supplement: Supplementary file 3 — Source Data [file 41467_2020_15762_MOESM3_ESM.zip › SOURCE DATA/Fig.3 - SiN optical measurements and dataprocessing/SiN_fit_Cov.png]

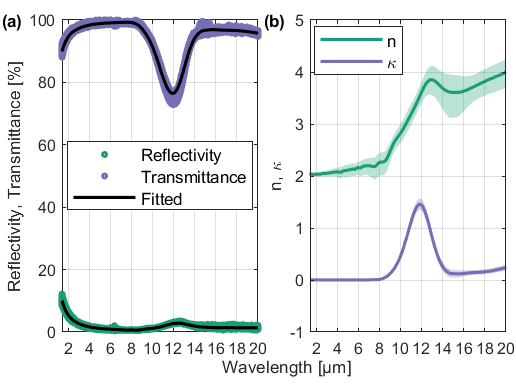

Supplement: Supplementary file 3 — Source Data [file 41467_2020_15762_MOESM3_ESM.zip › SOURCE DATA/Fig.3 - SiN optical measurements and dataprocessing/SiN_fit_MSE.png]

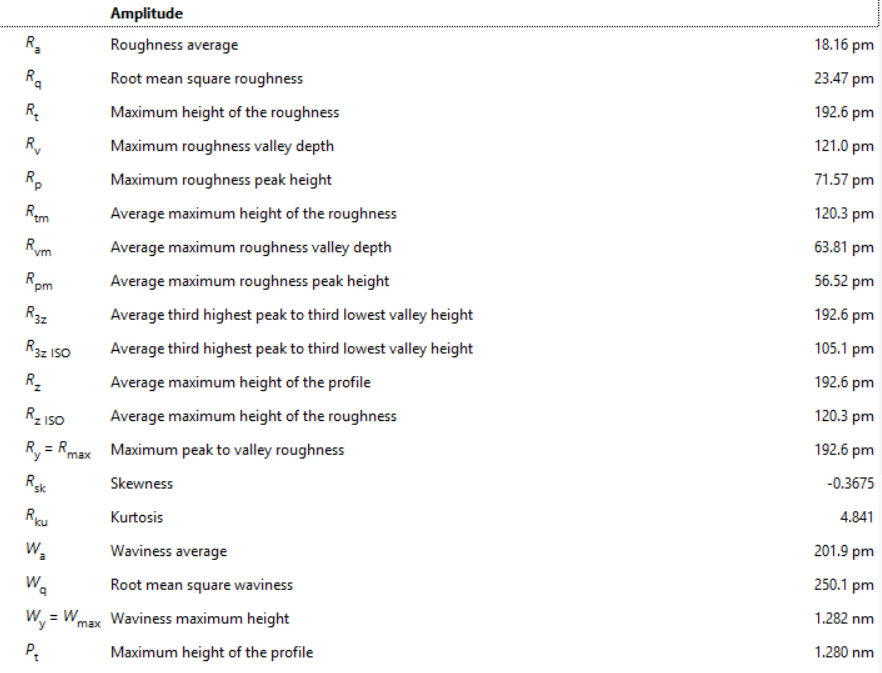

Supplement: Supplementary file 3 — Source Data [file 41467_2020_15762_MOESM3_ESM.zip › SOURCE DATA/Supplement Fig.1 - AFM Analysis/Roughness-Evaluation-bare-SiN.PNG]

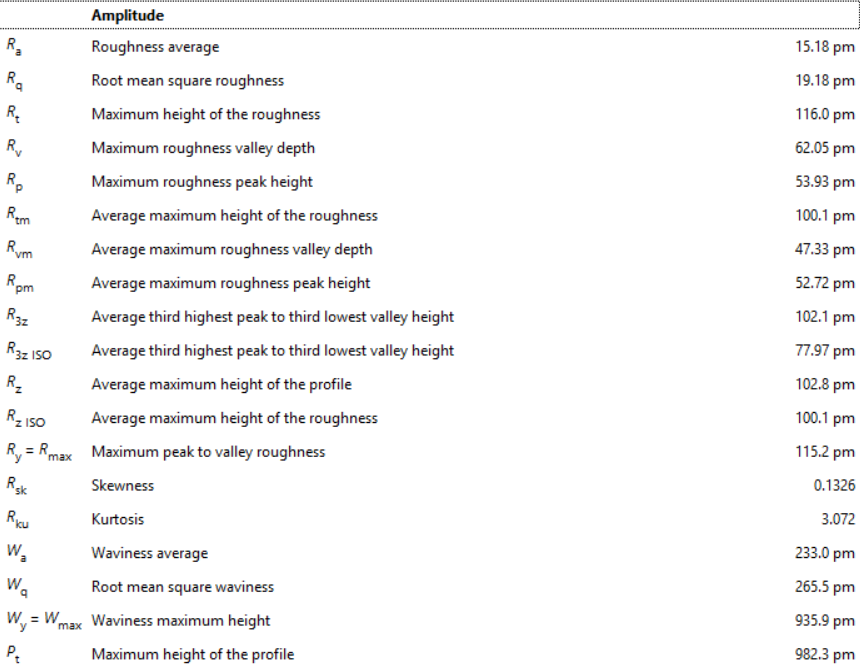

Supplement: Supplementary file 3 — Source Data [file 41467_2020_15762_MOESM3_ESM.zip › SOURCE DATA/Supplement Fig.1 - AFM Analysis/Roughness-Evaluation-UTMF.PNG]

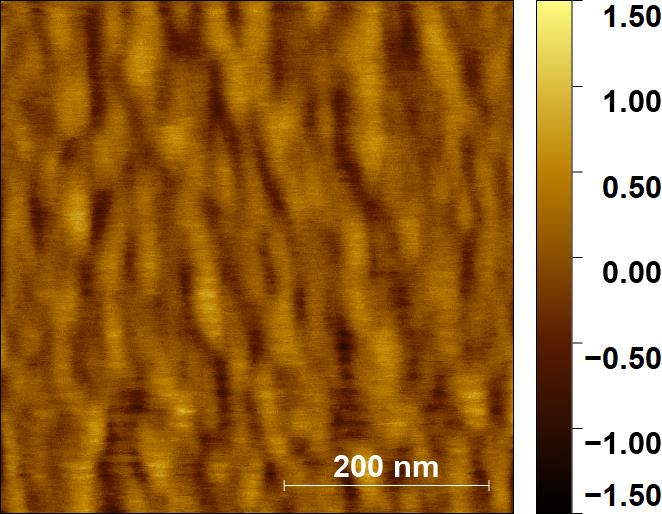

Supplement: Supplementary file 3 — Source Data [file 41467_2020_15762_MOESM3_ESM.zip › SOURCE DATA/Supplement Fig.1 - AFM Analysis/SiN-Ref-AFM.jpg]

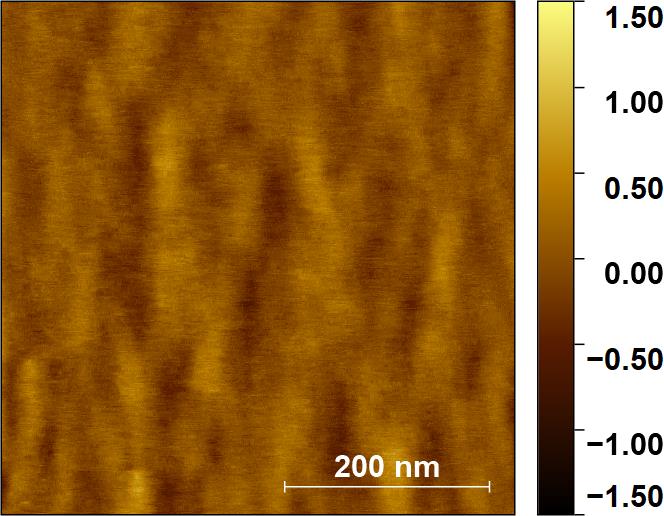

Supplement: Supplementary file 3 — Source Data [file 41467_2020_15762_MOESM3_ESM.zip › SOURCE DATA/Supplement Fig.1 - AFM Analysis/UTMF-2nm-AFM.jpg]

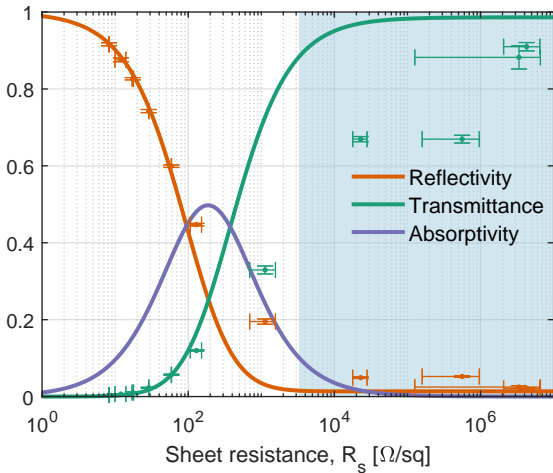

Supplement: Supplementary file 3 — Source Data [file 41467_2020_15762_MOESM3_ESM.zip › SOURCE DATA/Supplement Fig.2 - RTA vs Rs/RTA_vs_Rs.pdf]

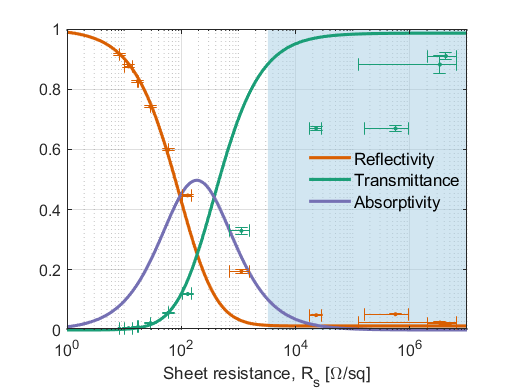

Supplement: Supplementary file 3 — Source Data [file 41467_2020_15762_MOESM3_ESM.zip › SOURCE DATA/Supplement Fig.2 - RTA vs Rs/RTA_vs_Rs.png]

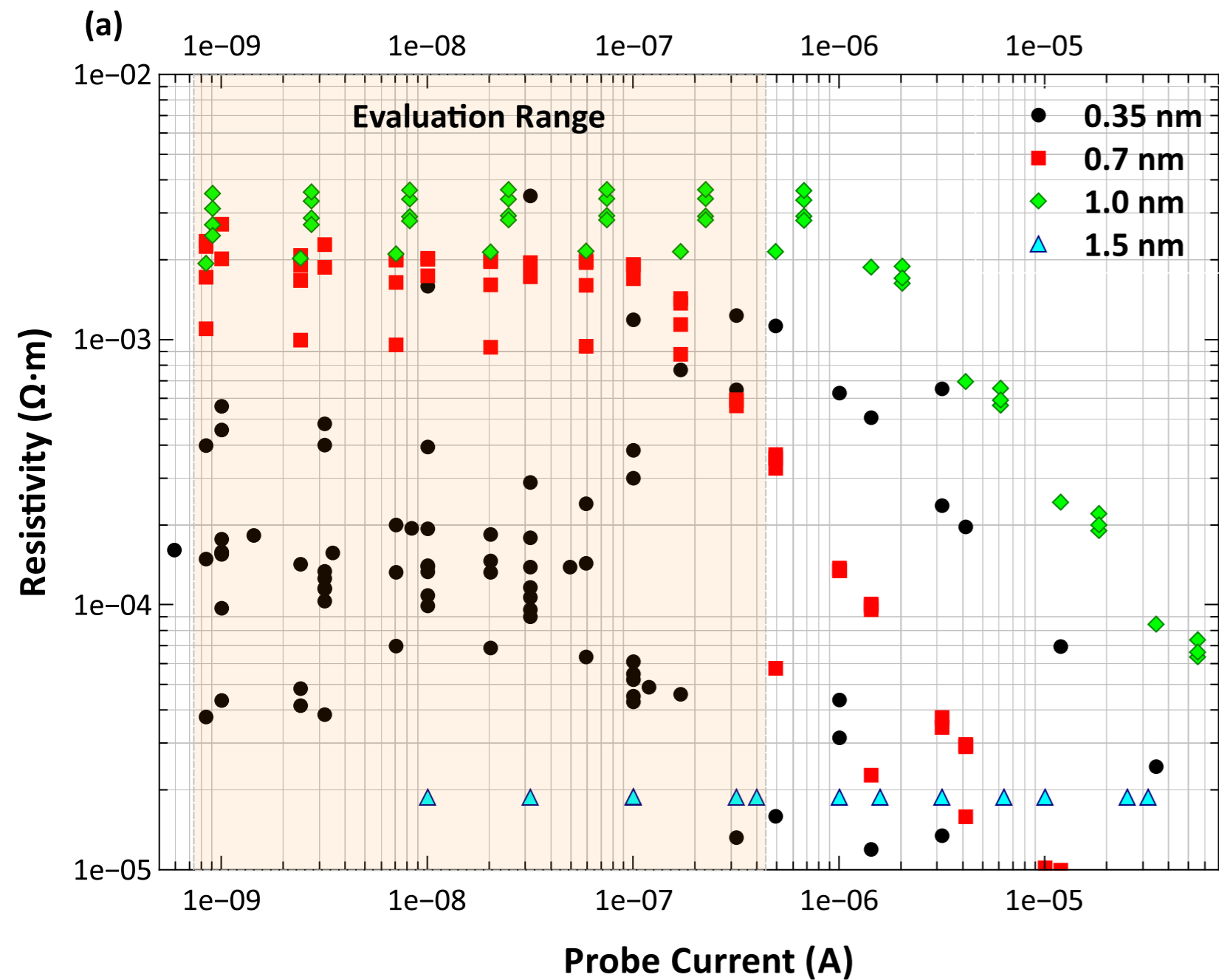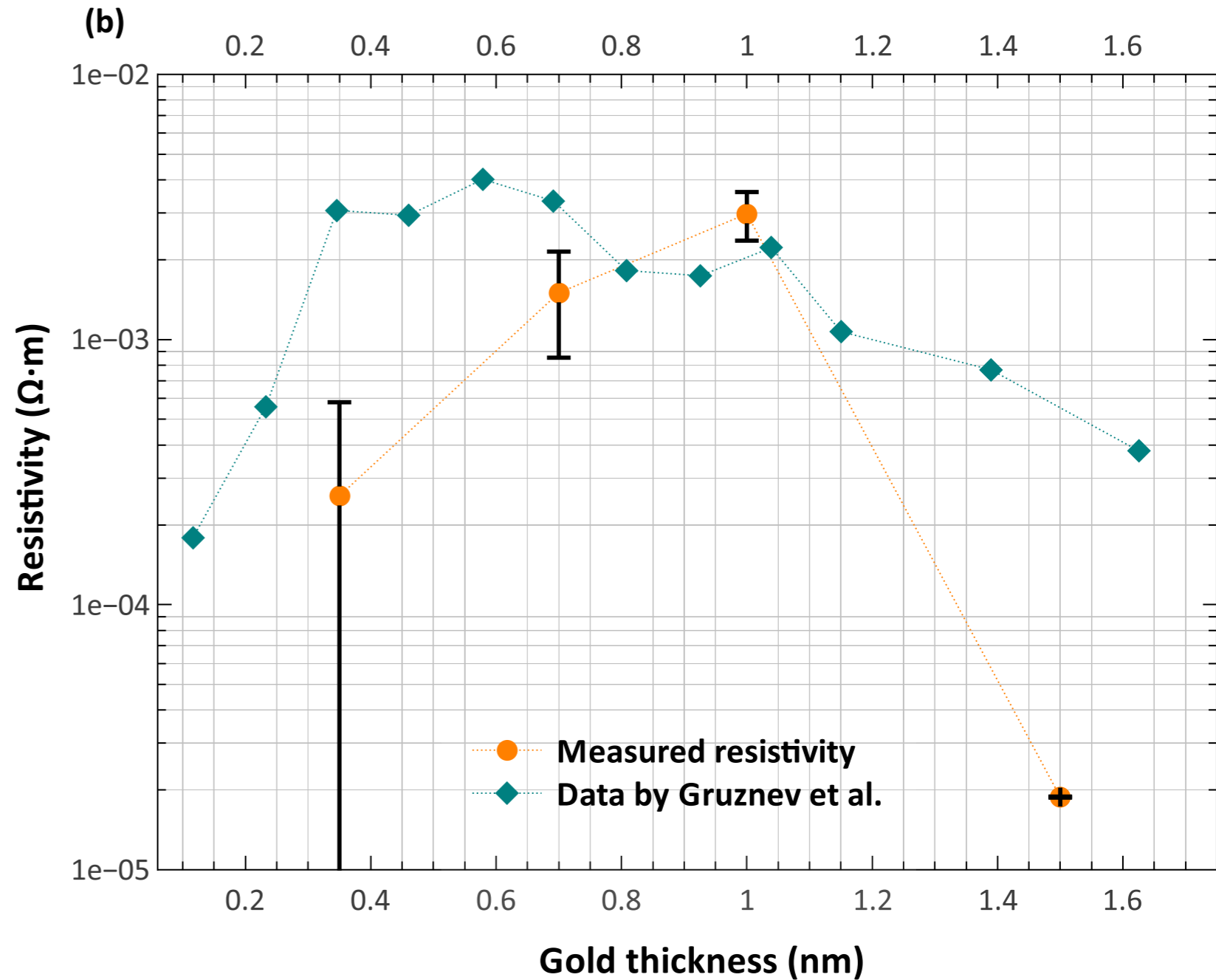

Supplement: Supplementary file 3 — Source Data [file 41467_2020_15762_MOESM3_ESM.zip › SOURCE DATA/Supplement Fig.3 - Resistivity below percolation/Resistivity-Comparison.pdf]
